# Larger Collections and Increased Grants: The Total of £70,600

**Published:** 1915-08-07

**Authors:** 


### THE WAR AND THE HOSPITAL SUNDAY FUND.

### Larger Collections and Increased Grants.

### THE TOTAL OF £70,600.

A meeting of the Council of the Metropolitan Hospital Sunday Fund was held at the Mansion House on Thursday, July 29. In the absence of the Lord Mayor the chair was taken by Sir C. Ernest Tritton, Bart., Vice-Chairman. Twenty-two members of the Council were present.

### The Distribution and the War.

The Chairman expressed the regret of the meeting that the Lord Mayor was not able to be present; he was presiding over a meeting at the Guildhall, where the Freedom of the City of London was being presented to the Prime Minister of Canada, that great Dominion which had responded so loyally to the Empire's call, and had sent such splendid troops to help the Empire's cause. The Report presented to the Council, he thought, would be considered a very satisfactory one. In spite of the endless appeals which had been put forward in the last twelve months, the Fund would be able to distribute, as Sir William Church would presently state, about £10,000 more than was available last year. That showed that the hospitals of London still had a very warm place in the hearts of the inhabitants of the Metropolis, and that this year they were more anxious than ever to do their best on Hospital Sunday, because they realised how splendidly there had been treated, within the walls of the hospitals, large numbers of brave wounded soldiers who had returned from the Front. All must have realised what a grand thing it had been to have these hospitals ready to take in and treat such large numbers of men when they had been maimed in battle. The thanks of the Council were due to the clergy and other ministers of all denominations for the way in which they pleaded the cause of Hospital Sunday; and those thanks must be extended to the congregations for the most generous support which they gave in response to the appeals from the pulpits. Thanks were likewise due to those many private donors who had most generously sent in large sums and thus materially helped to swell the total. He also expressed appreciation to the Press for their helpful appeals.

#### The Special Effort.

Sir William Church, Bart., K.C.B., moved: "That the Report of the Committee of Distribution for the year 1915 be, and the same is hereby, approved, and that the awards thereby recommended be paid forthwith." There was little that he need say, as the Chairman had alluded to the most interesting fact that, owing to the special efforts put forward and the generous response, the Council had this year a larger sum to dispense than had been the case for a good many years. On looking back, he found that there had been larger sums than this year, though not much larger; and he thought the general feeling would be one of pleasure at the result, especially as it had been feared that this abnormal year would result in a diminished subscription.

Sir Dyce Duckworth, LL.D., seconded the resolution with much pleasure. At the meeting last November he ventured to forecast that the subscriptions this year would not materially fall off, in spite of the war; and it was a great satisfaction to him to find that such an idea had been justified. The motion was carried unanimously.

The Rev. A. A. Harland proposed: "That the best thanks of the Council be and are hereby accorded to the Committee of Distribution for the care they have bestowed upon the preparation of the awards." They all, as a Council, felt deeply indebted to the gentlemen who had undertaken such an arduous duty; and they were only too thankful that such a large sum had been subscribed, considering the times in which we were now living.

Mr. John Robartes seconded, and it was carried.

#### The Value of Newspapers.

The Rev. Hardy Harwood moved: "That the thanks of the Council be and are hereby given to the editors of newspapers who have pleaded the needs of the hospitals, and advocated the cause of this Fund." There had been, he said, considerable discussion of late as to the value of newspapers in certain directions, but he thought all would agree that, however great might be the differences of opinion on certain questions, the unanimity which was a characteristic of this Fund was reflected in the comments of the daily Press upon its work. If it should happen that an editor was in doubt as to what he should fill a column with, he could not do better than plead the cause of the hospitals, and such help of the kind as had already been given had undoubtedly contributed to the splendid result achieved this year. He hoped a similar kindness could be relied upon in the future.

Lord Meath, in seconding, said he felt that if it had not been for the newspapers it would have been impossible to obtain the sums which the Fund had been able to distribute; and this year they had pointed out that if there was one thing which the public ought to support, apart from actual war work, it was the hospitals, because they were doing such an enormous amount of work for the wounded soldiers. If that fact were carefully impressed on the minds of the public, he believed next year's total would surpass that which the Council were now so grateful over. In Dublin, where he had to do with a similar institution, there had been a like experience; for whereas for a number of years there had been a steady diminution of funds, the collection this year was the largest they had ever experienced. Much of that result, he felt sure, was due to the efforts of the Dublin Press. He hoped newspapers everywhere would make it clear that, whatever else might suffer, the hospitals should not be allowed to do so. The resolution was carried.

### "Hospital-Sunday and You."

The Chairman moved: "That the best thanks of the Council be and are hereby given to Mr. Pett Ridge for writing, and to Mr. Fred Pegram for illustrating, the booklet entited 'Hospital-Sunday and You,' which undoubtedly contributed to the success of the Fund's appeal this year." The idea originated with the Secretary, Mr. James, who asked Mr. Pett Ridge to write the book, while the Assistant-Secretary, Mr. Lander, asked Mr. Pegram to draw the illustrations. His own view was that the brochure was admirably written and artistically illustrated. Prebendary Eardley-Wilmot seconded, and it was unanimously carried.

Alderman Sir Marcus Samuel praised the personal work of Sir William Church, Chairman of the Committee of Distribution, which had now become a science. Not the slightest favouritism was shown and absolute justice was done to every claiming institution. The Committee over which Sir William Church presided entered minutely into every detail when elucidation was required, and the public had such confidence in the method of administration that he felt sure support would continue to be forthcoming for the necessary upkeep of the hospitals.

Thanks to Sir Ernest Tritton.

The Rev. E. S. Hilliard proposed a cordial vote of thanks to Sir Ernest Tritton for presiding and for the work he did for the Fund. He desired it should go beyond mere formality, for no one except those in the office knew how much time and thought Sir Ernest Tritton devoted to the cause, and he was a very strong factor in its success. When the Fund had the misfortune to lose its revered Secretary, Sir Ernest did much in the reorganisation of the office, and so a special meed of thanks was due to him.

Mr. F. C. Carr-Gomm seconded, cordially supporting the words of the proposer, and it was carried by acclamation.

The Chairman, in assuring the meeting of his appreciation, said it was to him a labour of love, for he felt very keen about the Fund's continued success. He also spoke highly of the genial and successful work of the Secretary and his assistant. He felt that the present meeting was no ordinary one, owing to the great generosity of the response which had been made to the appeal for support to the hospitals of the Metropolis.

